# Gene expression of inflammasome components in peripheral blood mononuclear cells (PBMC) of vascular patients increases with age

**DOI:** 10.1186/s12979-015-0043-y

**Published:** 2015-10-06

**Authors:** Xiaoyu Wu, Maani Hakimi, Markus Wortmann, Jian Zhang, Dittmar Böckler, Susanne Dihlmann

**Affiliations:** Department of Vascular and Endovascular Surgery, University of Heidelberg, Im Neuenheimer Feld 110, 69120 Heidelberg, Germany; Department of Vascular & Thyroid Surgery, The First Hospital of China Medical University, Shenyang, China; Vaskuläre Biomaterialbank Heidelberg, VBBH, Heidelberg, Germany

**Keywords:** Vascular disease, Inflammation, Aging, AIM2, NLRP3, Atherosclerosis

## Abstract

**Background:**

Chronic low-grade inflammation is considered a driver of many age-related disorders, including vascular diseases (inflammaging). Inhibition of autophagic capacity with ageing was postulated to generate a pro-inflammatory condition via activation of inflammasomes, a group of Interleukin-1 activating intracellular multi-protein complexes. We thus investigated gene expression of inflammasome components in PBMC of 77 vascular patients (age 22–82) in association with age.

**Findings:**

Linear regression of real-time qRT-PCR data revealed a significant positive association of gene expression of each of the inflammasome components with age (Pearson correlation coefficients: *AIM2*: r = 0.245; *P* = 0.032; *NLRP3*: r = 0.367; *P* = 0.001; *ASC (PYCARD):* r = 0.252*; P* = 0.027; *CASP1*: r = 0.296; *P* = 0.009; *CASP5*: r = 0.453; *P* = 0.00003; *IL1B*: r = 0.247; *P* = 0.030). No difference in gene expression of *AIM2, NLRP3, ASC CASP1*, and *CASP5* was detected between PBMC of patients with advanced atherosclerosis and other vascular patients, whereas *IL1B* expression was increased in PBMC of the latter group (*P* = 0.0005).

**Conclusion:**

The findings reinforce the systemic pro-inflammatory phenotype reported in elderly by demonstrating an increased phase-1 activation of inflammasomes in PBMC of vascular patients.

**Electronic supplementary material:**

The online version of this article (doi:10.1186/s12979-015-0043-y) contains supplementary material, which is available to authorized users.

## Findings

### Background

The concept of inflammaging implies that a low-grade pro-inflammatory status, predisposing the organism to chronic diseases, appears during the ageing process [1]. A number of reports have provided experimental evidence for this hypothesis and remodeling of the immune system was postulated to drive many age-related disorders [2]. In addition, altered function of innate immune cells, in particular changes in monocytes and macrophages, have been described in mice [3] and humans [4]. Clinically, aging is associated with increases of circulating IL-18 and IL-6 plasma levels, whereas IL-1β is undetectable [5, 6].

During aging, injuries, necrotic cell death and metabolic cell stress accumulate thereby increasing the risk of chronic inflammation [7, 8]. Dead cells and their metabolites are usually cleared by autophagy though macrophages. A disturbed interplay between autophagy and the inflammasomes was recently postulated to link inflammaging with vascular and other pathologies [9]. The inflammasomes are a family of multiprotein complexes for danger signal recognition that are induced by pathogens or cell debris (damage associated molecular patterns, DAMPS) [10, 11]. Expression levels of the inflammasome components are generally low and require a two phase induction: A priming phase stimulates gene expression of inactive precursors before a second signal can stimulate assembly of the multiprotein complex consisting of sensor proteins, the adaptor protein apoptosis-associated speck-like protein with a caspase activation and recruitment domain (ASC) and inflammatory caspases (Caspase-1 or Caspase-5). Upon assembly, the caspases are enzymatically cleaved by the complex resulting in their active form, which then stimulates enzymatic cleavage of Interleukin-1β (IL-1β) and IL-18 from inactive precursors [10]. Depending on the initial sensor, several subfamilies are distinguished: The NOD-like receptors (NLR), including NLRP3 and others, act as sensors for intracellular damage associated signals such as cholesterol crystals, nanoparticles, reactive oxygen species and others. The DNA sensors, including Absent in Melanoma 2 (AIM2) act as sensors for intracellular dsDNA [10].

Given the recently demonstrated involvement of inflammasome activation in vascular disease [12–14] and its putative role in inflammaging, we here addressed gene expression of inflammasome components in PBMC of vascular patients in association with age.

## Materials and methods

Venous blood was taken from 77 vascular patients on the day of their hospitalization according to the standard operating procedures of the Vascular Biobank Heidelberg (VBBH). All patients gave their written informed consent to the study, which was approved by the ethical committee of the University of Heidelberg (S-301/2013 and S-412/2013). Patients’ characteristics are described in Additional file 1: Table S1.

PBMC and plasma were separated according to standard procedures (for details see Additional file 2), RNA extraction and reverse transcription were performed as described earlier ([13] and Additional file 2). Quantitative analysis of gene expression was performed by real time PCR and relative expression was determined by using individual standard amplification curves of each transcript relative to the corresponding mean expression of three reference transcripts (*GAPDH* plus *B2M* plus *ACTB*).

Active, cleaved IL-1β (p17) in plasma was quantified by ELISA, Caspase-1 protein was detected by Western blotting (for details see Additional file 2).

## Results and discussion

**Fig. 1:**
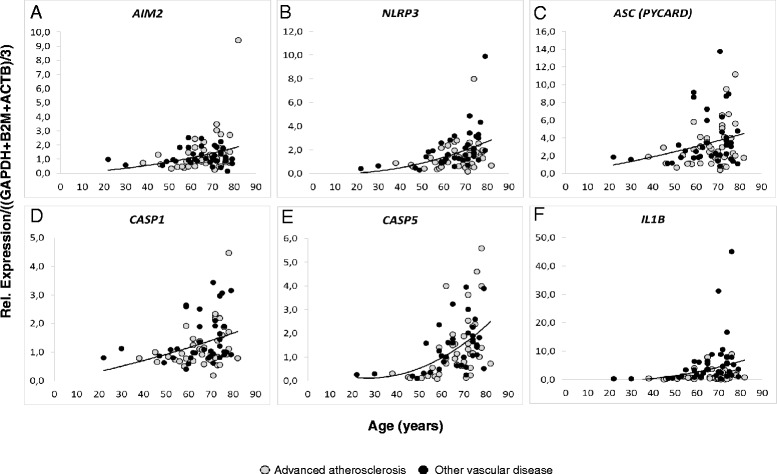
Best fit regression of PBMC gene expression with age of the patients. Dot plots represent the rel. expression of AIM2 (**a**), NLRP3 (**b**), ASC (**c**), CASP1 (**d**), CASP5 (**e**) and IL1B (**f**) normalized to the mean rel. expression of three reference genes (*GAPDH, B2M, ACTB*) in PBMC in relation to the age of the patient. Further details are described in materials and methods of the online data supplements

As shown in Fig. 1, best fit regression revealed a close association of *AIM2, NLRP3, ASC, CASP1, CASP5,* and *IL1B* gene expressions with age of the patients. The strongest correlation was found for *CASP5* with a correlation coefficient of 0.468 (*P* = 0.00003). Regression coefficients and p-values of the linear regression analysis are summarized in Table 1.

**Table 1:** Linear regression analysis of gene expression with age of the patients

	Pearson correlation Coefficient	Standard Error	95 % CI	*P-*value
*AIM2*	0.245	0.074	0.094 - 0.369	0.032
*NLRP3*	0.367	0.054	0.254 - 0.477	0.001
*ASC(PYCARDN*	0.252	0.080	0.114 - 0.412	0.027
*CASP1*	0.296	0.089	0.116 - 0.483	0.009
*CASP5*	0.453	0.069	0.320 - 0.570	0.00003
*IL1B*	0.247	0.373	0.150 - 0.373	0.030

**Fig. 2:**
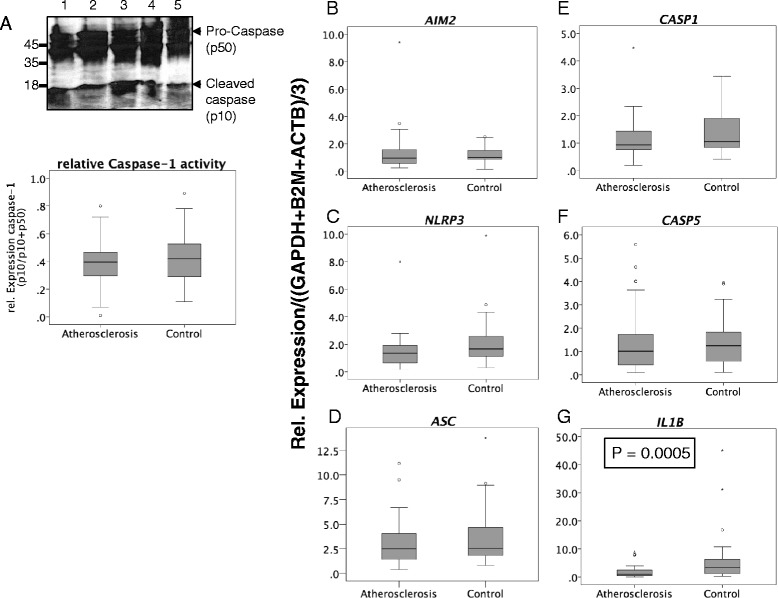
**a** Examples of a western blot showing expression of Pro-Caspase-1 and its cleavage products in PBMC from five different patients, and comparison of the p10/(p10 + p50) ratios according to vascular disease. Twenty μg of each PBMC lysate was loaded per lane. Details for detection are described in the Additional file 2. **b** to **g** Comparison of inflammasome gene expression in PBMC according to vascular disease. Gene expressions in PBMC of patients with advanced atherosclerosis (carotid stenosis, peripheral artery disease) were compared with PBMC gene expression in patients with other vascular diseases. Details of patients characteristics are described in materials and methods of the online data supplements

To examine, whether the increased expression of inflammasome components resulted in increased activation of the inflammasome multiprotein complex, we next determined the amount of its effector proteins, activated caspase-1 (p10) and activated Il-1β (p17) . Active IL-1β was detected in plasma of 3 out of 30 patients with abdominal aortic aneurysm and in plasma derived from one patient with an aortic dissection (data not shown). Western blotting of PBMC lysates showed cleaved, active caspase-1 (p10) together with pro-Caspase-1 (p50) in all samples (Example in Fig. 2a). Neither the IL-1β plasma levels nor the ratio of Caspase-1 p10/p10 + p50, indicating relative activity of the inflammasome complex, were associated with age (data not shown).

To adjust for vascular diseases, such as atherosclerosis, that might interfere with inflammasome activation [12, 15], inflammasome gene expression and activities in the group of patients with advanced atherosclerosis (carotid stenosis, peripheral artery disease and arterial stenosis; *n* = 39) were compared with those derived from patients with other vascular disease (aortic aneurysms, other; *n* = 38). As shown in Fig. 2, *AIM2, NLRP3, ASC, CASP1, and CASP5* gene expressions did not differ between the groups (Fig. 2b-f). In contrast, *IL1B* gene expression was significantly higher in the control group compared to the atherosclerosis group (*P* = 0.0005; Fig. 2g and Additional file 1: Table S1). This unexpected finding may be ascribed to the composition of the control group which contained many samples from patients with aortic aneurysms. Locally increased gene expression of *IL1B* is known to be increased in AAA tissue samples [16]. Whether PBMC from AAA-patients do also express increased levels of *IL1B* and other inflammasome components is currently under our investigation and will be published in a separate study. No difference in the relative levels of active Caspase-1 (p10) could be detected between the groups (Fig. 2a and Additional file 1: Table S1).

Despite some indications from animal experiments [17], it is currently unknown, whether systemic inflammasome activation is associated with human ageing. Our data present clear evidence for this hypothesis by demonstrating, that gene expression of *AIM2, NLRP3, ASC, CASP1, CASP5,* and *IL1B* in PBMC of vascular patients increases with age. Future studies on inflammasome gene expression of PBMC in healthy people of different ages will be necessary to demonstrate whether this phenomenon applies to ageing in general, although the definition of “healthy” might be difficult in individuals above 70 years.

Regardless of the limitation to vascular patients, our findings reinforce results from previous studies, describing age-associated mechanisms that are required for priming of inflammasome components. For example, constitutive transcriptional activity of NF-κB, which is necessary for induction of *NLRP3* and *IL1B* gene expression*,* has been demonstrated in aged tissues and organisms [18–20]. In addition, increased levels of the AIM2 protein in human fibroblasts were associated with senescence and increased production of IL-1β [21].

Since phase-1 activation was not accompanied by increased IL-1β and Caspase-p10 levels in our study, we conclude that full inflammasome assembly and activation, requiring a second signal [10], do not occur with a higher frequency in PBMC of the elderly. This is in line with previous reports, demonstrating that PBMC of young and older individuals do not differ in Il-1β release after stimulation with LPS in vitro [4].

Instead, our data point to an age-associated shift towards a preactivated first line of defense by innate immunity. Given a systemic increase in priming of inflammasome genes through constitutive gene expression in PBMC with ageing, the threshold for full inflammasome activation by the second signal derived from local DAMP recognition in different tissues might be reached earlier and predispose individuals to vascular and other chronic diseases. Cummulative evidence supports the existence of such age-associated changes in the cellular components of the innate immune system [2, 4]. However, as yet, we cannot deduce from our data, whether the increased inflammasome gene expression results from a general activation across all PBMC or from an altered PBMC composition, i.e. an increase in monocyte numbers, as reported previously [22]. Further investigations, addressing this question are currently under examination. In addition, our ongoing studies aim to elucidate the interaction of primed PBMC with vascular tissues and their impact on vascular disease progression. The reason for the increased gene expression of inflammasome components in PBMC during ageing is unknown so far. Besides DAMPS and pathogens serving as immunomodulators, the sympathethic nervous system was recently described to affect the functions of both innate and adaptive immune cells via the β-adrenergic receptor (β-AR) [23, 24]. It remains to be shown, whether ageing-associated changes in β-AR function contribute to altered inflammasome gene expression in PBMC.

In summary, the present study adds important findings to the concept of inflammaging by demonstrating an age-dependent increase in activation of innate immunity via systemic priming of inflammasomes in vascular patients.

## Additional files

Additional file 1: Table S1. Comparison of age, sex and gene expression in PBMC from patients with advanced atherosclerosis and PBMC from other vascular patients. (DOCX 16 kb) Additional file 2:
**Materials and methods.** (DOCX 24 kb)

## Abbreviations

AIM2: Absent in Melanoma 2
ASC: Apoptosis-associated speck-like protein with a caspase activation and recruitment domain
CASP: Caspase
DAMP: Damage-associated molecular pattern
IL: Interleukin
IL-1β: Interleukin-1β
IL-18: Interleukin-18
IL-6: Interleukin-6
NLRP3: NOD-like receptor protein 3
PBMC: Peripheral blood mononuclear cells
