# Supplementary material for: Gene expression of inflammasome components in peripheral blood mononuclear cells (PBMC) of vascular patients increases with age
Source: Immun Ageing. 2015 Oct 6;12:15. doi: 10.1186/s12979-015-0043-y (PMC4596365; doi:10.1186/s12979-015-0043-y)
Supplement: Additional file 1: Table S1. — Comparison of age, sex and gene expression in PBMC from patients with advanced atherosclerosis and PBMC from other vascular patients. (DOCX 16 kb) [file 12979_2015_43_MOESM1_ESM.docx]

**Supplemental results**

**Supplemental table 1:**

**Patients characteristics and comparison of gene expressions in PBMC from patients with advanced atherosclerosis and PBMC from other vascular patients**

|  | **Atherosclerosis**  n = 39 | | **Control**  n = 38 | ***P*-value** |
| --- | --- | --- | --- | --- |
| Age at blood donation (ys) | 69 (38-82) | 69.5 (22-79) | | 0.894 |
| Sex, n (% female) | 8 (20.51%) | 6 (15.79%) | | 0.769 |
| Relative gene expression of *AIM2* | 0.94 (0.24-9.42) | 1.00 (0.14-2.52) | | 0.511 |
| Relative gene expression of *NLRP3* | 1.35 (0.17-7.99) | 1.67 (0.29-9.90) | | 0.057 |
| Relative gene expression of *ASC(PYCARD)* | 2.49 (0.39-11.17) | 2.54 (0.81-13.74) | | 0.421 |
| Relative gene expression of *CASP1* | 0.94 (0.19-4.47) | 1.06 (0.41-3.44) | | 0.125 |
| Relative gene expression of *CASP5* | 1.01 (0.09-5.58) | 1.25 (0.11-3.96) | | 0.415 |
| Relative gene expression of *IL1B* | 0.96 (0.04-8.99) | 3.44 (0.29-45.06) | | 0.0005 |
| Protein level of active Caspase-1 p10/(p10+p50) | 0.40 (0.01-0.80) | 0.42 (0.11-0.89) | | 0.512 |

Data was represented as median ± (minimum-maximum) or number (%). Age and relative gene expressions of *AIM2, NLRP3, ASC(PYCARD), CASP1 CASP5* and *IL1B* were analyzed by Mann-Whitney *U* test. Chi squared test was used for the analysis of sex difference.
