# Supplementary material for: Gene expression of inflammasome components in peripheral blood mononuclear cells (PBMC) of vascular patients increases with age
Source: Immun Ageing. 2015 Oct 6;12:15. doi: 10.1186/s12979-015-0043-y (PMC4596365; doi:10.1186/s12979-015-0043-y)
Supplement: Additional file 2: — Materials and methods. (DOCX 24 kb) [file 12979_2015_43_MOESM2_ESM.docx]

**Materials and Methods**

**Blood samples and patients characteristics**

Venous blood was taken from 77 vascular patients on the day of their hospitalization according to the standard operating procedures of the Vascular Biobank Heidelberg (VBBH). All patients gave their written informed consent to the study, which was approved by the ethical committee of the University of Heidelberg (S-301/2013 and S-412/2013). Patients’ characteristics are described in supplementary table 1.

Supplementary table 1: Patients characteristics

| Age | µ = 65,6 *ys* | range 22-82 |
| --- | --- | --- |
| Male  Female | N = 63  N = 14 | age range 22-82 ys  age range 30-78 ys |
|  |  |  |
| *Vascular disease:* |  |  |
| Aortic aneurysm | N = 30 |  |
| Carotid stenosis (symt. or asympt.) | N = 23 |  |
| Peripheral artery disease (stage II-IV) | N = 14 |  |
| Aneurysms (not aorta) | N = 3 |  |
| Arterial stenosis (A. brachiocephalicus, A. mesenterica superior) | N = 2 |  |
| Thrombosis (A. poplitea) | N = 1 |  |
| Aortic dissection | N = 1 |  |
| Aortic stenosis (Lerich syndrome) | N = 1 |  |
| Angina abdominalis | N = 1 |  |
| Peripheral malfomation | N = 1 |  |

**PBMC preparation**

Within 2-4 hours after blood donation, up to 8 ml anti-coagulated blood was separated in Leucosep tubes (Greiner Bio-one GmbH, Frickenhausen, Germany) prefilled with 3 ml Ficoll Paque (GE Healthcare, Uppsala, Sweden), according to the instructions of the manufacturer. Upon centrifugation, plasma supernatant was harvested and stored at -80°C until further analysis. The enriched cell fraction containing peripheral blood mononuclear cells (PBMC) was harvested and washed twice in 10 ml phosphate-buffered saline (PBS). Cell pellets were shock-frozen for cryopreservation at -80°C until further usage.

**RNA isolation, cDNA synthesis and RT-qPCR**

Total RNA was extracted from cryopreserved PBMC pellets using RNeasy mini Kit (Qiagen, Hilden, Germany) according to the manufacturer’s instructions. For expression analysis, 0.2 µg of the total RNA was reverse transcribed using oligo-dT primers and SuperSript III reverse transcriptase (Life Technologies, Carlsbad, CA, USA). For real-time PCR, Power SYBR Green master mix (Life Technologies) was added to appropriate cDNA samples and specific primers (supplementary table 2) as previously described (Dihlmann et al., 2014). Quantitative analysis of gene expression was performed with a StepOne Plus Real time PCR System (Life technologies) at a C_t_ threshold of 0.34 for all samples. Relative expression was determined from cycle thresholds (C_T_) by using individual standard amplification curves of each transcript (supplementary table 2) relative to the corresponding mean expression of three reference transcripts (*GAPDH* plus *B2M* plus *ACTB*).

Supplementary table 2: gene names, transcripts, primers and standard amplification curves used for qPCR analysis

| Gene name | Transcript No. (ENSEMBLE) | Primer forward (5’->3’) | Primer reverse (5’->3’) | Standard curve |
| --- | --- | --- | --- | --- |
| *AIM2* | AIM2-001 ENST00000368130 | CTGTTAGACCAGTTGGCTTG | AGCTGACATCTGGAGTTCATAGC | C_T_ = -1,1871x + 26,46 |
| *ACTB* | ACTB-001 ENST00000075624 | CACCATGTACCCTGGCATTG | AGTACTTGCGCTCAGGAGG | C_T_ = -1,2738x + 17,15 |
| *ASC (PYCARD)* | PYCARD-001 ENST00000247470 | AAGCCAGGCCTGCACTTTAT | CTGGTACTGCTCATCCGTCA | C_T_ = -1,2973x + 18,52 |
| *B2M* | B2M-001 ENSG00000166710 | CCGTGTGAACCATGTGACTT | ATGCGGCATCTTCAAACCTC | C_T_ = -1,2973x + 18,52 |
| *CASP1* | CASP1-001 ENST00000436863 | CCACAATGGGCTCTGTTTTT | CATCTGGCTGCTCAAATGAA | C_T_ = -1,4771x + 22,29 |
| *CASP5* | CASP5-001 ENST00000260315 | TCATTTGAAGTTCCACAGGCTA | TGCCTGTGGTTTCATTTTCA | C_T_ = -1,0686x + 27,95 |
| *GAPDH* | GAPDH-001 ENST00000229239 | GGCTGCTTTTAACTCTGGTA | CTTGACGGTGCCATGGAATT | C_T_ = -1,2804x + 18,22 |
| *NLRP3* | NLRP3-001 ENST00000348069 | CCCAGGGATGAGAGTGTTGT | CAAGGAGATGTCGAAGCAGC | C_T_ = -1,2407x + 27,44 |
| *IL1B* | IL1B-001 ENST00000263341 | CTGAAAGCTCTCCACCTCCA | CCAAGGCCACAGGTATTTTG | C_T_ = -1,3147x + 26,18 |

**IL-1β ELISA**

Active, cleaved IL-1β (p17) in plasma was quantified by the Duo-Set ELISA Development system for human IL-1β (R&D Systems Europe, Abington, UK) according to the recommendations of the manufacturer. Briefly, 100 µl of undiluted plasma was loaded on 96 well plates pre-coated with IL-1β capture antibody. After washing, detection was performed using a human IL-1β detection antibody, Streptavidin-HRP and substrate solution. The optical density was determined in a microplate reader at 450 nm with a correction at 560 nm. Concentration of IL-1β was calculated using a standard curve.

**Caspase-1 Western Blotting**

PBMC samples were lysed in RIPA buffer and homogenized by ultrasound. Twenty µg of each lysate was separated by SDS-PAGE an blotted onto nitrocellulose (0,2 µm). Detection of Caspase p50 and p10 was performed over night with Caspase-1 p10 rabbit pAb (Santa Cruz Biotechnology, Heidelberg, Germany) diluted 1 : 200 in Tris buffered saline containing 1 % non-fat dry milk). Anti-rabbit IgG HRP-linked secondary antibody (Cell Signaling Technology) and Western-lightning plus ECL (Perkin Elmer, Waltham, MA, USA) were used for detection. For relative quantification, signals of equal exposure times were densitometrically analyzed with ImageJ and normalized to corresponding GAPDH signals.

**Statistical analysis**

All data was analyzed by IBM SPSS version 21. ANOVA was used for the analysis of relative gene expressions. Pearson linear correlation was used for the analysis of association between age and gene expression levels. For comparison of gene expressions between patients with advanced atherosclerosis and controls, the median, minimum and maximum were determined and data groups were analyzed by the Wilcoxon-Mann-Whitney Test for unpaired samples. P-values below 0.05 were interpreted as significant.
